# The Role of Ghrelin in Neuroprotection after Ischemic Brain Injury

**DOI:** 10.3390/brainsci3010344

**Published:** 2013-03-19

**Authors:** Sarah J. Spencer, Alyson A. Miller, Zane B. Andrews

**Affiliations:** 1 School of Health Sciences and Health Innovations Research Institute (HIRi), RMIT University, Melbourne, VIC 3083, Australia; 2 Department of Pharmacology, Faculty of Medicine, Monash University, Melbourne, VIC 3800, Australia; E-Mail: alyson.miller@monash.edu; 3 Department of Physiology, Faculty of Medicine, Monash University, Melbourne, VIC 3800, Australia; E-Mail: zane.andrews@monash.edu

**Keywords:** ghrelin, neurodegeneration, apoptosis, inflammation

## Abstract

Ghrelin, a gastrointestinal peptide with a major role in regulating feeding and metabolism, has recently been investigated for its neuroprotective effects. In this review we discuss pre-clinical evidence suggesting ghrelin may be a useful therapeutic in protecting the brain against injury after ischemic stroke. Specifically, we will discuss evidence showing ghrelin administration can improve neuronal cell survival in animal models of focal cerebral ischemia, as well as rescue memory deficits. We will also discuss its proposed mechanisms of action, including anti-apoptotic and anti-inflammatory effects, and suggest ghrelin treatment may be a useful intervention after stroke in the clinic.

## 1. Introduction

The search for neuroprotective treatments after ischemic brain injury has so far proved remarkably unsuccessful. Indeed, recombinant tissue plasminogen activator (rt-PA), first introduced in the 1980s [1], is currently the only useful treatment for ischemic stroke. Although rt-PA effectively restores blood flow to the brain it cannot directly target the fundamental mechanisms of ischemic injury. Furthermore, owing to the narrow therapeutic window (less than 4.5 h), only a small proportion of stroke patients actually receive rt-PA [2]. With ischemic stroke being the second leading cause of death and disability worldwide [3], there is a clear need for more effective therapies. As will be discussed below, emerging evidence suggests that ghrelin may be one such potential therapy. 

Ghrelin is a 28 amino acid peptide that is principally synthesized in the gut, but is also expressed in a variety of other tissues [4,5]. It was first identified in 1999 as a stimulator of growth hormone release [5]. Ghrelin exists in the plasma in a des-acylated and an acylated form, the latter of which is the result of post-translational octanoylation of pro-ghrelin by the enzyme ghrelin-*O*-acyltransferase (GOAT) [6]. Acylated ghrelin is the natural ligand of the growth hormone secretagogue receptor type 1a (ghrelin receptor, GHS-R1a) through which growth hormone release is stimulated [5,6,7]. 

Since this initial description, research has attributed many other functions to the peptide, including its well-known role in feeding and metabolism [8,9,10,11]. Under situations of food deprivation, acylated ghrelin signals through the hypothalamus to promote feeding and energy conservation [10,12]. Ghrelin also has a number of significant extra-hypothalamic neuronal functions, including in reward and motivation [13,14], learning and memory [15], and stress [16,17]. For instance, we have recently shown ghrelin attenuates the hypothalamic-pituitary-adrenal axis response to acute stress, probably by acting on the anterior pituitary to stimulate adrenocorticotropic hormone release and thus enhance glucocorticoid negative feedback [16]. 

The GHS-R1a is expressed throughout the brain including in feeding and metabolism-associated areas like the arcuate nucleus (ARC) [18,19,20], as well as regions important in memory (Cornu Ammonis (CA)2, CA3, dentate gyrus of the hippocampus) and reward (ventral tegmental area, substantia nigra pars compacta (SNpc), dorsal raphe) [19,21,22]. The GHS-R1a is a g-protein coupled seven transmembrane receptor. Ghrelin activates the GHS-R1a leading to stimulation of the phospholipase C (PLC)/protein kinase C (PKC)/inositol trisphosphate (IP3) pathway that triggers IP3-dependent calcium release from intracellular stores. This intracellular calcium coupled with calcium entering the cell via voltage-gated L-type calcium channels stimulates the GHS-R1a’s downstream responses [23,24,25]. In addition to its ghrelin-dependent effects, the GHSR has high constitutive activity in the absence of the ligand [26]. It is also able to dimerize with other receptors such as the dopamine receptor subtype 2 to modulate dopamine signaling [27], and the melanocortin-3 receptor to modulate melanocortin signaling [28]. Ghrelin itself is also able to cross the blood brain barrier [5,18,29,30], making the ligand and receptor ideally placed to play an integral role in these diverse functions. As discussed below, evidence now suggests ghrelin may also have a role in neuroprotection in the setting of neurodegenerative disease and ischemic injury.

## 2. Ghrelin’s Role in Neuroprotection

### 2.1. Parkinson’s and Alzheimer’s Diseases

Evidence for a role for ghrelin in neuroprotection came initially from findings that the peptide is able to inhibit apoptosis in cardiomyocytes [31], and protect these cells from myocardial ischemia-reperfusion injury [32,33]. These findings have since been extended to a role for ghrelin in neuroprotection in Parkinson’s (PD) and Alzheimer’s (AD) diseases [34]. Thus, endogenous and exogenous ghrelin have been shown to protect dopamine neurons of the SNpc in a 1-methyl-4-phenyl-1,2,5,6-tetrahydropyridine (MPTP)-induced mouse model of PD [35,36,37]. It also protects against dopamine depletion in the striatum [36]. In this PD model, ghrelin protects the brain by upregulating uncoupling protein 2 resulting in enhanced mitochondrial respiration and a reduction in reactive oxygen species production [36]. Ghrelin also reverses the pro-apoptotic effects of MPTP and enhances the firing rate of the dopamine neurons to enhance dopamine availability during degeneration [36]. Interestingly, recent evidence suggests ghrelin production may be reduced in PD patients [38], raising the possibility that a reduction in ghrelin-mediated neuroprotection may contribute to increased vulnerability of dopaminergic neurons. 

With respect to AD, it is also noteworthy that plasma levels of ghrelin naturally decrease with age (in AD and in age-matched non-dementia subjects) [39] but AD is further associated with reduced brain GHS-R1a levels and reduced levels of brain GOAT [40]. Exogenous ghrelin is able to rescue memory deficits in mice given amyloid beta oligomers into the hippocampus. It also reduces the amyloid beta oligomer-induced microgliosis and neuronal loss in this region, and prevents amyloid beta oligomer-associated synaptic degeneration [41]. *In vitro*, ghrelin is able to limit tau hyperphosphorylation and increase glucose uptake in hippocampal neurons via a mechanism that involves the phosphatidylinositol 3-kinase (PI3K)/Akt/glycogen synthase kinase-3 beta (GSK-3B) pathway [42].

### 2.2. Cerebral Ischemia

#### 2.2.1. Observations of Neuroprotection

It is also now evident that ghrelin can be a powerful neuroprotective agent in experimental models of cerebral ischemia. One of the first studies in this field revealed synthetic ghrelin (GHS-hexarelin) reduces injury to the cerebral cortex, hippocampus, and thalamus after neonatal hypoxia-ischemia [43]. As with AD, the neuroprotective effect here is associated with phosphorylation of Akt and GSK3B, indicating the PI3K pathway is involved. 

Early studies using models of adult stroke examined the effects of exogenous ghrelin administered immediately after either forebrain or focal ischaemia-reperfusion injury. Thus, Liu and colleagues [44] found ghrelin given i.p. daily for three days after a transient ischemia-reperfusion injury (four vessel occlusion) in the rat significantly increased the number of surviving neurons in the CA1 region of the hippocampus and significantly decreased the number of TUNEL-positive neurons in this region. Exogenous ghrelin also improves neurological deficit, infarct size, and survival of cortical neurons in rodents after transient focal ischemia-reperfusion (middle cerebral artery occlusion; MCAO) [45,46]. Again, similar to in PD models, its mechanism of action appears to be inhibition of pro-apoptotic pathways [45,46]. These findings are crucial from a clinical perspective. As with PD and AD, ghrelin levels are reduced in clinical stroke populations. In particular, ghrelin levels have been reported to be lower in male patients after cardioembolic stroke compared with the healthy controls [47]. Given the apparent neuroprotective properties of ghrelin, restoring ghrelin levels after stroke is therefore likely to have significant beneficial outcomes. We also suggest measuring ghrelin levels after stroke could be used as a diagnostic tool to predict prognosis.

#### 2.2.2. Mechanisms of Neuroprotection

There are three principal types of cell death induced by cerebral ischemia and hypoxic injury; apoptosis, necrosis, and autophagy [48]. In cases of focal ischemia, such as with an MCAO in rodents, or a focal stroke in humans, the major mechanism of cell death within the ischemic core is necrosis, and this occurs immediately after the insult [49]. The more delayed cell death that occurs in the ischemic penumbra (3–24 h and beyond) occurs via principally apoptotic and autophagic mechanisms [49], against which ghrelin may be useful. In cases of global cerebral ischemia, such as occurs in the brain after a cardiac arrest, the principal mechanism of cell death appears to be necrosis [50], and ghrelin may be less useful in these cases. However, we should note there is likely considerable overlap between these mechanisms of cell death. 

Apoptosis is a mechanism of cell “suicide” that is regulated by specific signaling pathways and can occur by caspase-dependent and caspase-independent mechanisms [48]. Necrosis has been termed a “cellular catastrophe”, where the cell fails due to a deficiency of ATP. However, it now appears some necrotic events are a consequence of specific signaling pathways. Necroptosis is one example of a necrotic-type cell death process that is regulated by cell signaling [48,49]. Neuronal cell death after ischemia can also occur via autophagy (type II death); a cellular degradation process wherein the mitochondria remain intact and functional. Autophagy is a normal cellular process that is initially protective and can promote neuronal survival [51], but under extreme conditions degradation of macromolecules and organelles such as the Golgi apparatus, endoplasmic reticulum, and polyribosomes ensues [48]. 

##### 2.2.2.1. Ghrelin Protects against Apoptosis

**Figure 1:**
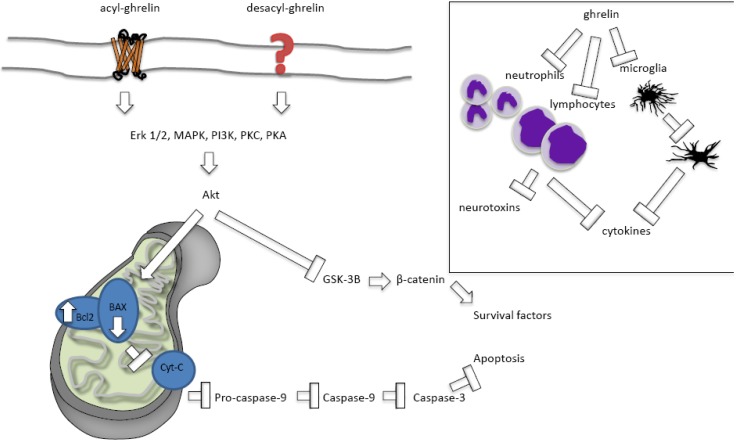
Ghrelin inhibits apoptosis and protects against inflammation. Ghrelin stimulates extracellular-signalling-regulated-kinase (ERK)1/2, mitogen-activated protein kinase (MAPK), protein kinase A (PKA), and protein kinase C (PKC) pathways to reduce activation of BAX, improve the Bcl2/BAX ratio and thus suppress apoptosis and improve cell survival. Inset; ghrelin inhibits neutrophil, lymphocyte, and microglial activation to suppress pro-inflammatory cytokine production and the secretion of inflammatory neurotoxins.

When a cell becomes sufficiently depleted of oxygen and nutrients, pro-apoptotic genes involved in cell death are activated. This activation leads to the stimulation of pro-apoptotic BAX and suppression of anti-apoptotic Bcl2. These proteins interact to regulate the permeability of the mitochondrial permeability transition pore [52,53,54,55]. When BAX is activated, cytochrome *c* is released from the mitochondria and interacts with Apaf-1 such that Apaf-1 forms a complex with pro-caspase 9, leading to activation of caspase-9 and -3. Mitochondrial dysfunction therefore plays an important role in cell survival. Evidence suggests ghrelin inhibits apoptotic mechanisms by activating the extracellular-signalling-regulated-kinase (ERK)1/2, mitogen-activated protein kinase, protein kinase A, and protein kinase C pathways [36,56]. The activation of these pathways is associated with reduced activation of BAX, an improved Bcl2/BAX ratio and suppression of apoptosis/improved cell survival (Figure 1). Ghrelin also suppresses apoptosis by increasing expression of mitochondrial uncoupling protein UCP2. UCP2 elevation effectively buffers production of reactive oxygen species, protecting the cell from oxidative stress and reducing apoptosis [36,57,58]. Protective increases in UCP2 with ghrelin have been observed in traumatic brain injury and PD models [36,58]. 

In the MPTP model of PD, ghrelin reverses the reduced expression of Bcl2 and increased expression of BAX associated with the MPTP to improve the Bcl2/BAX ratio and prevent apoptosis [35]. Similarly, in the MCAO (focal) and four vessel occlusion (forebrain) models of ischemia reperfusion, ghrelin suppresses the increase in the pro-apoptotic gene, Par-4, associated with the ischemia [44,59]. Ghrelin thus also improves the Bcl2/BAX ratio and inhibits cytochrome *c* release and caspase-3 activation [44,59]. The neuroprotective effects of ghrelin are also mediated by activation of PI3K/Akt. Stimulation of this pathway by ghrelin leads to phosphorylation and inactivation of GSK-3B and stabilization of beta-catenin [56,60]. Beta-catenin is then able to translocate to the nucleus and stimulate transcription of various cell survival factors [56].

This anti-apoptotic role for ghrelin is also seen after other neurodegenerative insults. Ghrelin ameliorates the reduction of pAkt and Bcl2 and attenuates the increase in caspase-3 expression in the caecal ligation and perforation model of sepsis. This action leads to a reduced cognitive deficit in this model [61]. Ghrelin also improves central outcomes after traumatic brain injury, suppressing caspase-3 expression and reducing cell death around the impact site [58]. *In vitro* studies also suggest an anti-apoptotic role for ghrelin, with ghrelin treatment of primary hypothalamic neurons inhibiting apoptosis after oxygen-glucose deprivation via rapid ERK1/2 activation [62]. 

##### 2.2.2.2. Ghrelin’s Effects on Necrosis and Autophagy

To our knowledge, unlike with apoptosis, there is no current evidence that ghrelin directly influences necrotic neuronal cell death. Ghrelin may protect against necrosis caused by persistent ischemia in musculoskeletal tissues, but it does so by up-regulating iNOS, which, in turn, improves the local microcirculation [63]. There is some evidence ghrelin may be able to encourage cell survival by stimulating autophagy in cardiomyocytes under simulated hypoxic conditions leading to a more efficient removal of damaged organelles and misfolded proteins [64]. However, this pathway has not yet been examined *in vivo* or in models of cerebral ischemia. Also, care must be taken with the interpretation of these results as extensive autophagy can be detrimental to the cell [48]. 

##### 2.2.2.3. Acylated *versus* Des-Acylated Ghrelin

It is interesting that ghrelin appears to be neuroprotective somewhat independently of acylation state [56]. Both ghrelin forms have been reported to prevent cell death and apoptosis in cultured neurons exposed to oxygen and glucose deprivation (an *in vitro* model of ischemia) [56,62], suggesting the neuroprotective actions of ghrelin *in vivo* are likely to occur through direct effects on neurons and independently of growth hormone release. Both acylated and des-acylated ghrelin also protect cortical neurons after transient focal ischemia-reperfusion (MCAO). In both cases, ghrelin can prevent apoptosis by suppressing the increase in expression of the pro-apoptotic gene Par-4 [56]. Acylated and des-acylated ghrelin also both improve the Bcl2/BAX ratio, and inhibit cytochrome *c* release and caspase-3 activation [59]. While it is clear the effects of acylated ghrelin are mediated by the GHS-R1a [56,59], des-acylated ghrelin does not activate GHS-R1a [34], and its neuroprotective effects are unaffected by the GHS-R1a antagonist d-Lys-3-growth hormone releasing peptide (GHRP)-6 [56]. The receptor through which des-acylated ghrelin mediates its neuroprotective effects remains to be determined. The GHS-R1a is certainly important for ghrelin’s neuroprotective actions, however. For example, in the MPTP model of PD, both ghr−/− and GHSR−/− mice have greater loss of dopamine neurons in the SNpc than wild type mice [36]. Similarly, expression of the GHS-R1a is reduced in the brain [45] and spinal cord [65] by ischemia-reperfusion and exogenous ghrelin treatment is able to reverse this effect, leading to an improved Bcl2/BAX ratio [45]. Of potential importance, the GHS-R1a has considerable constitutive activity in the absence of the ligand [26] and may significantly contribute to limiting apoptosis after ischemia even when ghrelin is low. 

##### 2.2.2.4. Ghrelin Protects against Inflammation

In the hours to days after an ischemic event, the degree of inflammation plays a significant role in the severity of the injury [66]. In the early stages of ischemia-reperfusion, neutrophils and lymphocytes are recruited to the injury site and these are mostly neurotoxic, secreting inflammatory mediators such as cytokines and contributing to brain injury [67]. Microglia and astrocytes are then activated and secrete pro-inflammatory cytokines and other factors, such as inducible nitric oxide synthase (iNOS), that are also cytotoxic [68]; although microglia also play a protective role in removing cytotoxic debris at some stages of the inflammation [66,69]. 

Another mechanism by which ghrelin may improve cell survival after ischemia is therefore by suppressing inflammation (Figure 1). Exogenously applied ghrelin successfully suppresses inflammation in many models of pathology, including sepsis [70], non-alcoholic fatty liver disease [71], burn-induced multiple organ injury [72], traumatic brain injury [73], and ischemia [46]. For instance, after subarachnoid haemorrhage, ghrelin can suppress the release of the pro-inflammatory cytokines tumour necrosis factor (TNF)α and interleukin (IL)-1β to improve outcomes [74]. Ghrelin suppresses TNFα and IL-6 induced by traumatic brain injury [73]. Likewise, ghrelin reduces the serum TNFα and myeloperoxidase activity induced by ischemic-reperfusion spinal cord injury [65].

Ghrelin’s neuroprotective role in PD is partially due to its anti-inflammatory effects. Thus, ghrelin is able to reduce MPTP-associated microglial activation in the SNcp and striatum as well as reduce the expression of TNFα, and IL-1β mRNA, and iNOS activation in the ventral midbrain to improve neuronal survival in these regions [37]. These anti-inflammatory effects are potentially GHS-R1a-mediated (*i.e.*, due to acylated ghrelin and not des-acylated ghrelin) as the antagonist, d-Lys-3-GHRP-6, attenuates the effect [37].

Ghrelin also extends this anti-inflammatory function to cerebral ischemia-reperfusion injury. Ghrelin treatment in rats after an MCAO reduced MCAO-induced neutrophil trafficking, TNFα, IL-6, matrix metalloproteinase 9, and nNOS, as well as apoptosis [46]. This treatment was associated with reduced infarct size, reduced neurological deficit, and improved 7-day survival. In this case it appears ghrelin’s mechanism of action is at least partially vagally mediated, as prior vagotomy blunted this neuroprotective effect [46]. A role for the vagus has also been suggested for ghrelin’s action in traumatic brain injury [75]. Ghrelin, thus, may be useful as an anti-inflammatory agent in cases of neurodegenerative disease. 

## 3. Clinical Considerations

Experimental evidence in animal models clearly suggests ghrelin may be a useful therapeutic after cerebral ischemia. This role may prove to be all the more crucial when we consider the ghrelin system appears to be altered in the setting of known stroke risk factors such as aging, obesity, and hypertension. For example, during the normal aging process there is a reduction in the sensitivity to the metabolic effects of ghrelin [76,77,78]. This insensitivity may be compounded by an aging-related decline in circulating ghrelin levels [39]. Obesity is associated with a central resistance to the metabolic effects of ghrelin [79], and ghrelin levels are reduced in obese and overweight humans [80]. Similarly, low ghrelin levels have also been reported to be independently associated with the development of type two diabetes and elevated blood pressure [81], and the Arg51Gln (rs34911341) single-nucleotide polymorphism of ghrelin is linked to an increased risk of hypertension, and is associated with low ghrelin levels [82]. Circulating ghrelin is also lower in patients who have experienced stroke than in the general population when controlling for factors such as age and obesity [47]. Collectively, these findings raise the intriguing possibility that low ghrelin levels *per se* may be a risk factor for stroke, and imply that ghrelin might not only be an effective intervention therapy after a stroke has occurred, but also an approach to prevent a stroke from occurring in the first place. There is therefore a strong rationale to evaluate whether increasing ghrelin levels and/or improving sensitivity to circulating ghrelin during aging is an effective strategy to prevent ischemic stroke damage. Of importance, experimental evidence indicates ghrelin can influence the sensitivity of the brain to its actions [62,83]. Specifically, studies have shown that GHS-R1a expression in the brain is increased following either central or peripheral administration of ghrelin to rodents [62,83]. Thus, sensitivity to circulating ghrelin could be improved during stroke risk factors (and after stroke) by “simply” increasing circulating ghrelin levels through ghrelin supplementation. 

An important consideration with using ghrelin as a therapeutic is the timing of the dose. As suggested, restoring normal ghrelin levels in aging or other populations where ghrelin is low may be a viable therapy, and in this case chronic ghrelin treatment would be necessary. An alternative strategy is to employ a single or repeated supra-physiological dose as a therapeutic agent and the vast majority of preclinical studies have tested single doses of exogenous ghrelin at the time of injury (Table 1). Unfortunately, this is likely to be effective only in the 30 min or so after administration, as ghrelin’s half-life is very short (approximately 25–30 min in humans [84,85]). As such, a single dose at this time would likely influence early apoptosis and inflammation, but not affect the ongoing ischemic injury. Since brain damage after ischemic injury can continue to worsen for hours to days and weeks after the event depending upon the type of injury [86], chronic ghrelin treatment may be more appropriate after injury as well as a prophylaxis. In either case, such treatments need to be considered carefully and further research is needed. For instance, studies have shown glutamatergic neurotransmission is necessary for ghrelin’s effects on mesolimbic dopaminergic reward pathways [87] and that ghrelin stimulates memory formation in a glutamate-dependent manner [15]. However, excessive glutamate release can lead to excitotoxicity [88], which would obviously be detrimental after ischemic injury. The circadian timing of the ghrelin administration must also be considered, as evidence from the PD model suggests exogenous ghrelin may only be effective if the subject is fasted [36]. 

When evaluating the overall usefulness of ghrelin as a therapeutic for cerebral injury after ischemia, it is also important to consider the potential long-term consequences on body homeostasis. For example, exogenous acylated ghrelin would be expected to act at the GHS-R1a to stimulate appetite, food intake, and weight gain. While this may be a problem in the general population, it may actually be of benefit in those recovering from stroke as weight loss after stroke has been linked with a worse long-term prognosis [89]. Thus, ghrelin as a stroke therapy could have an additional beneficial effect by preventing age-related malnutrition and frailty, as well as weight loss after stroke. An alternative option in those where weight gain is a concern is also to employ the des-acylated form of ghrelin. Des-acylated ghrelin does not act at GHS-R1a and does not have the same metabolic effects as the acylated form. It is, however, effective at preventing neurodegeneration and improving outcomes after ischemic damage [56]. Further research will be necessary to determine its mechanism of action and effects on other neuronal functions such as reward and stress, but treatment with des-acylated ghrelin is nonetheless a useful potential option.

**Table 1:** Effects, and associated mechanisms, of ghrelin on outcomes after cerebral ischemia. All studies were conducted in rats except where indicated. Arrows are relative to vehicle-treated ischemic groups.

Ischemic injury	Dose	Timing	Effect	Mechanism of action	Reference
Oxygen-glucose deprivation in cultured cells	Acylated and unacylated ghrelin, 100 nM each	Pretreatment for 24 h	↓ cell death (both)	↓ apoptosis	[56,62]
Doxorubicin H9c2 cardiomyocytes	Total ghrelin, 1 µM	Co-treatment for 24 h	↓ cell death (both)	↓ apoptosis GHSR-1a-independent mechanism	[31]
4VO forebrain ischemia/reperfusion	Total ghrelin, i.p., 0.4 mg/kg	Daily for 3 days post injury	↑ cell survival CA1 hippocampus	↓ apoptosis	[44]
MCAO Focal ischemia/reperfusion	Total ghrelin, i.v., 10 pmol/kg	Immediately post injury	↓cortical neuron injury	↓ apoptosis ↑ expression of GHSR-1a	[45]
MCAO	Total ghrelin, i.v., ~7 pmol/kg	Immediately post injury infusion for 1 h	↓ neurological deficit, ↓ infarct size at 24 h and 7 days	↓ apoptosis ↓ inflammation	[46]
MCAO	Total (80 µg/kg) or desacyl (160 µg/kg), i.p.	30 min prior to injury and immediately post	↓ cortical neuron injury (both)	↓ apoptosis GHSR-1a-independent mechanism	[59]
Neonatal hypoxia-ischemia	GHS-hexarelin, icv, 1 µg in 5 µL	Immediately post injury	↓ cortical, hippocampal, thalamic injury, ↔ striatum	↓ apoptosis	[43]
Spinal cord ischemia/reperfusion	Total ghrelin, i.p., 100 µg/kg	Ischemia onset	↑ neurological scores	↓ apoptosis ↓ inflammation ↑ expression of GHSR-1a	[65]
Subarachnoid hemorrhage	Total ghrelin, i.p., 10 µg/kg/day	Immediately post injury and 24 h later	↑ neurological scores	↓ inflammation	[74]
Traumatic brain injury	Total ghrelin, i.v., 4, 8 or 16 nmol/rat	45 min post-injury	↓ cortical neuron injury ↓ behavioural deficits	↓ apoptosis ↓ inflammation	[73]
Traumatic brain injury	Total ghrelin, i.p., 10 µg/kg/dose	Immediately prior to and 1 h post injury	↓ cell death	↓ inflammation ↓ blood brain barrier permeability	[58]

## 4. Conclusions

The research reviewed here suggests the peptide hormone, ghrelin, may be an exciting novel candidate in our search for treatments for ischemic brain injury. It is clear ghrelin treatment can provide significant neuroprotection in a number of models of neurodegenerative disease and following ischemic brain injury. Indeed, ghrelin is effective in improving cell survival, reducing infarct size and rescuing memory in these models. It does this primarily by suppressing the apoptosis and inflammation associated with the ischemic injury. However, it remains to be determined whether ghrelin also modulates necrotic or autophagic mechanisms of cell death. Nonetheless, these exciting data collectively point to ghrelin potentially being very useful in the clinic as a neuroprotective treatment. 
